# Incidence and root cause analysis of near‐miss events in medical device use errors in intensive care units using Ishikawa diagram

**DOI:** 10.1111/jjns.70024

**Published:** 2025-09-15

**Authors:** Su Mi Seong, Hyeop Oh, Jae Suk Park, Su Hyun Bae, Ki Chang Nam, Sung Yun Park, Bum Sun Kwon, Bo Hae Kim

**Affiliations:** ^1^ Department of Otorhinolaryngology‐Head and Neck Surgery Dongguk University Ilsan Hospital Goyang Korea; ^2^ Department of Nursing Dongguk University Ilsan Hospital Goyang Korea; ^3^ Center for Medical Device Safety Information Monitoring Dongguk University Ilsan Hospital Goyang Korea; ^4^ Department of Medical Engineering Dongguk University College of Medicine Goyang Korea; ^5^ Department of Diagnostics, College of Korean Medicine Dongguk University Goyang Korea; ^6^ Department of Physical Medicine and Rehabilitation Dongguk University Ilsan Hospital Goyang Korea

**Keywords:** healthcare near misses, intensive care units, medical errors, patient safety, root cause analysis

## Abstract

**Aim:**

This study aimed to investigate the incidence of near‐miss events related to medical device use errors (MUEs) in intensive care units (ICUs) and to identify their root causes using the Ishikawa diagram.

**Methods:**

This observational study was conducted in a referral hospital ICU in South Korea between August and September 2023, involving 60 nurses (29 MICU, 31 SICU) who completed anonymized questionnaires on near‐miss events related to five commonly used medical devices. Root causes were analyzed with a modified Ishikawa diagram. Data were processed using SPSS software. Independent *t*‐tests, ANOVA, and Pearson correlation were used for continuous variables, while chi‐square and Fisher's exact tests were applied to categorical data. One‐way ANOVA identified major contributing factors.

**Results:**

Each participant experienced an average of 2.11 ± 12.53 near‐miss events per device per year, with the highest incidence in IV line sets. A positive correlation was found between near‐miss frequency and years of work experience. Root cause analysis (RCA) showed that the most common contributing factors were work environment factors, especially high patient load. The main contributing factors included chronic fatigue (personal factors), frequent device malfunctions (medical device usability factors), and insufficient education programs (unit communication and culture/education factors).

**Conclusions:**

The study highlights the importance of improving working conditions, updating outdated equipment, and strengthening educational programs to reduce MUEs and improve patient safety in ICUs.

## 1 INTRODUCTION

Since various types of medical devices are simultaneously applied to patients in intensive care units (ICUs) for administering drugs and monitoring patients' conditions (Lewandowska et al., 2020; Marshall et al., 2017; Tunlind et al., 2015), medical staff should be cautious of errors when using these devices. Efforts to reduce medical errors, including those related to medical devices, have been increasing because these events can be fatal to patients and significantly contribute to rising medical costs (Ahsani‐Estahbanati et al., 2022; Rodziewicz et al., 2018). According to the World Health Organization (WHO), in 2000, one‐third of the more than 90,000 medical device‐related errors reported in the United States were identified as medical device use errors (MUEs) (World Health Organization, 2010). MUEs refer to errors in which the outcomes of medical device usage differ from the intended outcome without any malfunction of the device (Levine et al., 2022). Although MUEs were previously dismissed as user faults, many factors—both individual and systemic, including workplace environment, institutional education programs, and safety culture—are now recognized as contributors to MUEs (Levine et al., 2022; Wiklund et al., 2016). Since many cases of MUEs are known to be preventable by managing predisposing factors, various factors leading to MUEs have been investigated (Levine et al., 2022; Rodziewicz et al., 2018; Wiklund et al., 2016).

The initial step for preventing MUEs is to assess the current status of their occurrences (Rodziewicz et al., 2018). However, clinicians suspect that MUEs are underreported due to concerns about potential blame, which hinders the identification of their causes and leads to improper prevention plans (Rodziewicz et al., 2018). According to Heinrich's law, events with no injuries—defined as “near‐miss events” in the medical field—are known to be more frequent and may precede actual injuries (Rodziewicz et al., 2018; Seta et al., 2007). Therefore, investigating the incidence of near‐miss events in MUEs (NM‐MUEs) that do not cause actual harm to patients may help estimate the true incidence of MUEs (Rodziewicz et al., 2018); therefore, medical staff can report these events relatively without concern about potential disadvantages. After identifying the status of MUEs, the next step in developing an error prevention plan involves identifying the causes and root causes of these events (Joint Commission, 2020; Kellogg et al., 2017; Rodziewicz et al., 2018; Singh et al., 2024).

Root cause analysis (RCA), a systematic approach to identify possible root causes of unexpected events, provides insights into the problems contributing to errors (Joint Commission, 2020; Kellogg et al., 2017; Rodziewicz et al., 2018; Singh et al., 2024). Therefore, RCA has been widely used to promote patient safety in the medical field by determining the root causes of medical errors (Joint Commission, 2020; Kellogg et al., 2017; Singh et al., 2024). Among various methods for RCA, the Ishikawa diagram, also known as a “fishbone diagram,” is a well‐known tool for brainstorming all possible contributing factors to identify the main potential causes among various factors (Wong et al., 2016). The problem to be investigated is placed in the “head” of the fish, and the contributing factors are arranged along the “bones” of the fish (Wong et al., 2016). Among these factors, the primary contributor is identified as the key factor influencing the problem.

This study aimed to investigate the incidence of NM‐MUEs of the most commonly used medical devices in the ICUs and identify their root causes using the Ishikawa diagram.

## 2 METHODS

### 2.1 Study design and participants

This observational study was conducted at a referral hospital in South Korea between August and September 2023. A total of 60 ICU nurses who use medical devices on patients participated in the study: 29 from the medical ICU (MICU) and 31 from the surgical ICU (SICU).

The committee for this study comprised six members from the Medical Device Safety Monitoring Center at our institution, which operates with the support of the National Institute of Medical Device Safety Information in South Korea. This committee included two physicians with over 10 years of clinical experience, two professors specializing in medical engineering, and two chief nurses from medical and surgical intensive care units. The members participated in all stages of the research, including nurse interviews, developing the study plan, designing the questionnaire, modifying the Ishikawa diagram, and conducting the survey.

### 2.2 Inclusion and exclusion criteria

The inclusion criteria were as follows: registered ICU nurses who used medical devices for patient care, those working in either the MICU or SICU of the study hospital, and individuals who agreed to participate voluntarily.

The exclusion criteria included incomplete or blank questionnaires and staff members not directly involved in the use of medical devices.

Candidates for medical devices were selected based on the data of device‐related errors reported to the Medical Device Safety Monitoring Center at our institution. Finally, five types of medical devices were determined to be studied through interviews with active nurses: (1) continuous saturation monitor system (SpO_2_ monitors), (2) continuous electrocardiogram monitor system (EKGs), (3) intravenous infusion sets (IV line sets), (4) infusion pumps, and (5) medical beds.

### 2.3 Data collection

The questionnaire, which included both quantitative and categorical items based on an Ishikawa diagram framework, was developed by a multidisciplinary expert committee to assess awareness and the frequency of near‐miss medical device errors. This study is based on a comprehensive literature review, institutional MUE data, and interviews with ICU nurses (Kellogg et al., 2017). A preliminary test was conducted to assess the clarity and relevance of the instrument, followed by revisions based on expert consensus.

To protect participant privacy and reduce the potential for blame related to near‐miss events, the questionnaire was designed to collect only minimal personal information. The collected information included gender, years of nursing experience, and the number of patients in charge. The questionnaire featured a “yes/no” question confirming whether participants were aware of MUEs and their adverse effects on patient safety, as well as a question assessing the importance of managing MUEs using a visual analog scale ranging from 0 to 10. The number of NM‐MUEs (number of events per year, NOE) experienced in a year when using each medical device was investigated, depending on recall.

A Cronbach's α value exceeding 0.7 indicates an acceptable level of reliability, suggesting that the internal consistency of the questionnaire is satisfactory (Barbera et al., 2020).

Data were collected over a 10‐day period between August and September 2023 in the MICU and SICU of a referral hospital in South Korea. The study focused on five medical devices commonly associated with near‐miss events in the ICUs.

Printed questionnaires were distributed by a research administrative staff member who was not involved in the study design or data analysis. Each envelope contained a notice informing participants that they could choose not to participate by either not submitting the questionnaire or returning it blank. Participants were asked to return the forms within 10 days using a locked collection box placed in a private location within the ICUs.

The criteria for data selection, including the inclusion and exclusion criteria, are described in Section 2.2.

### 2.4 Instruments

**FIGURE 1:**
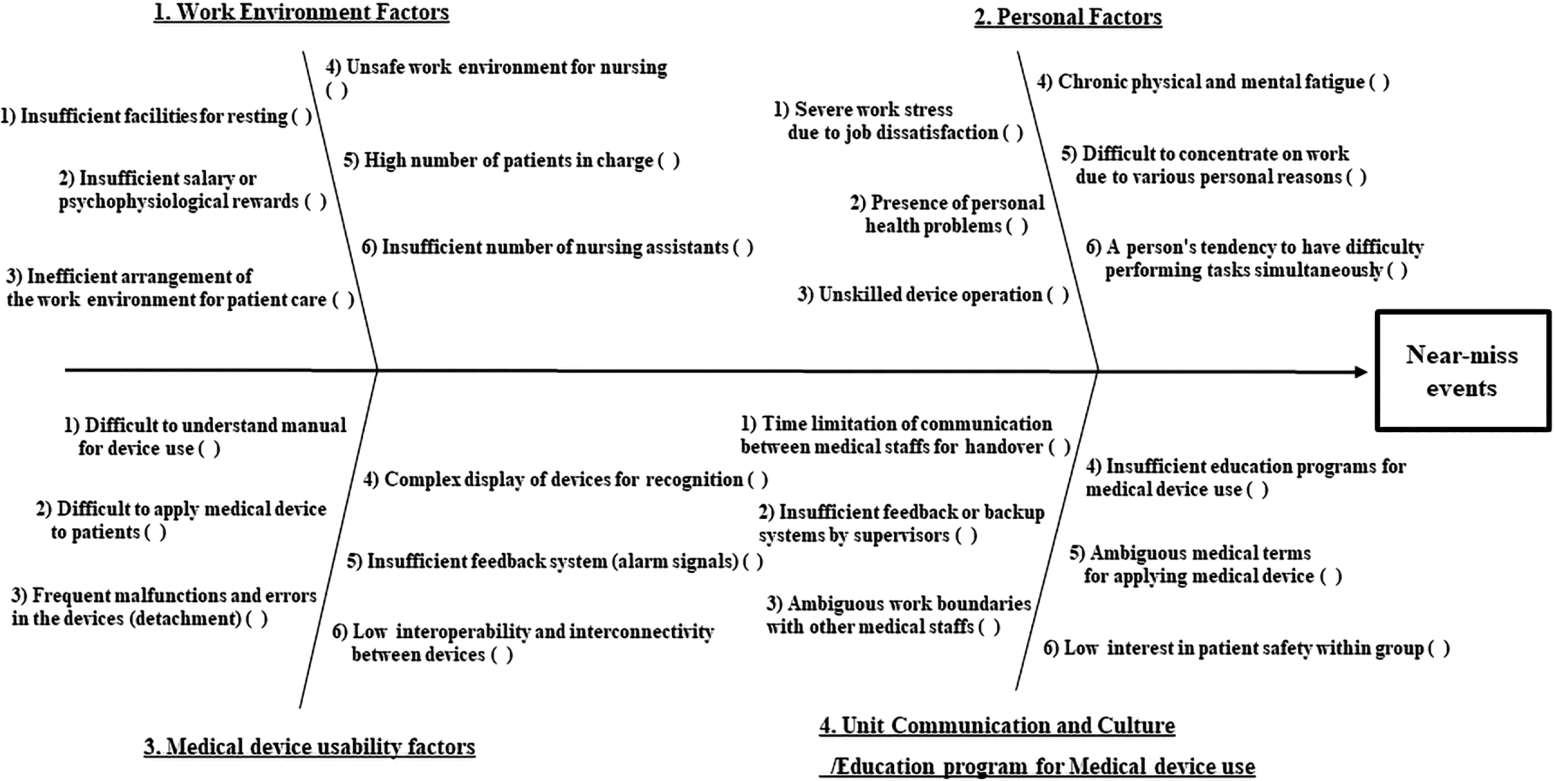
The Ishikawa diagram.

The Ishikawa diagram was designed by modifying the RCA framework recommended by the Joint Commission International, based on committee discussions (Joint Commission, 2020; Singh et al., 2024; Wong et al., 2016). In our study, the “fish head” of the diagram represents the “Near‐Miss Events,” and potential contributing factors are depicted in the “fish bone” structure. Main root causes were categorized into work environment, personal factors, medical device usability factors, and unit communication/education programs for medical device use (Figure 1) (Wong, 2011; Wong et al., 2016). Each main root cause contains six subcategories, and multiple selections are possible, with a score out of 6 points (Figure 1). The subcategories were collected from Food and Drug Administration (FDA) data on human factors that can affect medical device use and MUEs reported in our hospital, as well as nurse interviews, and ultimately determined through committee meetings (Johnson et al., 2007; Lee, 2021; Tanaka et al., 2010).

The results of the analysis combining the five medical devices were presented as “Total Medical Devices” (Total). To describe the specific findings obtained from the analysis of each device, a narrative review of the distinct RCA for each medical device was conducted.

### 2.5 Statistical analysis

We used SPSS software version 22.0 for analysis (IBM Corp., Armonk, NY). Questionnaires with incomplete responses were excluded prior to analysis. All analyses were conducted following assessment of normality using the Shapiro–Wilk test.

For continuous variables, an independent *T*‐test was utilized for demographics and the NOE‐associated factor analysis. Additionally, ANOVA was used to analyze the NOE on each device and to identify the main root cause categories in the Ishikawa diagram, both individually and in the combined “All Medical Devices” dataset. The Pearson correlation coefficient was used to assess the relationship between NM‐MUEs and continuous variables such as work experience, number of patients in charge, and importance of awareness.

For categorical variables, the chi‐square test was applied to demographics, while Fisher's exact test was used to compare frequencies among subcategories in the Ishikawa diagram.

### 2.6 Ethical considerations

This study was approved by the Institutional Review Board at the Dongguk University Ilsan Hospital (IRB No. DUIH 2023‐07‐007). All participants were informed, both in writing and verbally, that their participation was entirely voluntary, that they could withdraw from the study at any time without disadvantage, and that no personally identifiable information would be collected. Responses were anonymous, and strict confidentiality was maintained throughout the study. This study was conducted in accordance with the ethical principles of the Declaration of Helsinki.

## 3 RESULTS

### 3.1 Participant characteristics

A total of 58 out of 60 subjects completed the questionnaire (28 subjects affiliated with MICU and 30 subjects affiliated with SICU). There was no statistically significant difference in gender distribution, the average work experience, and the number of patients in charge between the subjects affiliated with MICU and SICU (Table 1). Except for one in 58 subjects, most of the subjects recognized that MUEs were a threat to patient safety (96.4%). The importance of MUEs prevention in patient safety was recognized as 8.30 ± 1.34 points out of 10 [Range: 6–10].

**TABLE 1:** Description of participants.

Characteristics	Total no. (%)	MICU no. (%)	SICU no. (%)	*p*‐value
Total participants	58 (100.0)	28 (48.2)	30 (51.7)	
Sex				.643
Male	19 (32.7)	10 (52.6)	9 (47.3)	
Female	39 (67.2)	18 (46.1)	21 (53.8)	
Work experience (ICUs)	5.45 ± 4.29	5.46 ± 4.21	5.48 ± 4.35	.997
Number of patients in charge	3.13 ± 0.52	3.28 ± 0.46	3.08 ± 0.36	.166
Recognition				.483
YES	57 (98.2)	27 (96.4)	30 (100.0)	
NO	1 (1.7)	1 (3.5)	0 (0.0)	
Importance of awareness (on a scale of 10)	8.30 ± 1.34	8.54 ± 1.32	8.18 ± 1.23	.331

Abbreviations: Importance of cognition, importance of awareness of medical device use errors; No., number; Recognition, recognition of the impact of analyzing medical device use errors on patient safety; Work experience (ICUs), work experience at intensive care units.

### 3.2 Incidences of near‐miss involving medical devices

**FIGURE 2:**
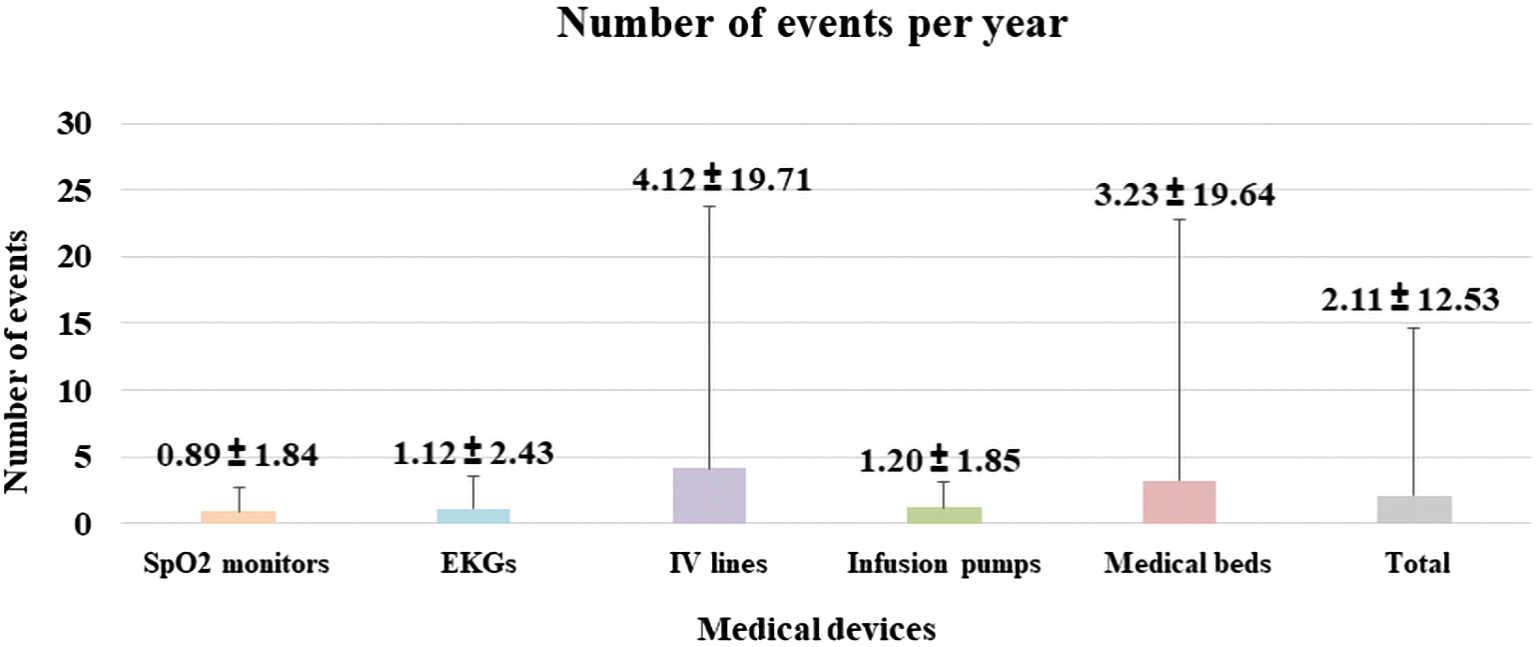
Number of events per year (NOE). Data are presented as mean ± standard deviation and was analyzed using one‐way ANOVA. SpO_2_ monitors, peripheral oxygen saturation monitors; EKC, electrocardiogram; IV line sets, intravenous fluid line set; Total, mean of all medical devices.

For five medical devices that were investigated in this study, each person experienced an average of 2.11 ± 12.53 [Range: 0–30] near‐miss events per device per year, with the highest incidence observed in IV line sets (4.12 ± 19.71) [Range: 0–30], followed by medical beds (3.23 ± 19.64) [Range: 0–30], infusion pumps (1.20 ± 1.85) [Range: 0–10], EKGs (1.12 ± 2.43) [Range: 0–10], and SpO_2_ monitors (0.89 ± 1.84) [Range: 0–10]. However, NOE of near‐miss among devices showed no significant difference (*p* = .533, Figure 2). In the Pearson correlation coefficient analysis conducted to identify the cause of high variation in standard variation, the total NOE per device exhibited a positive correlation with years of work experience (*r* = .351, *p* = .007).

### 3.3 Root cause analysis of total medical devices

**FIGURE 3:**
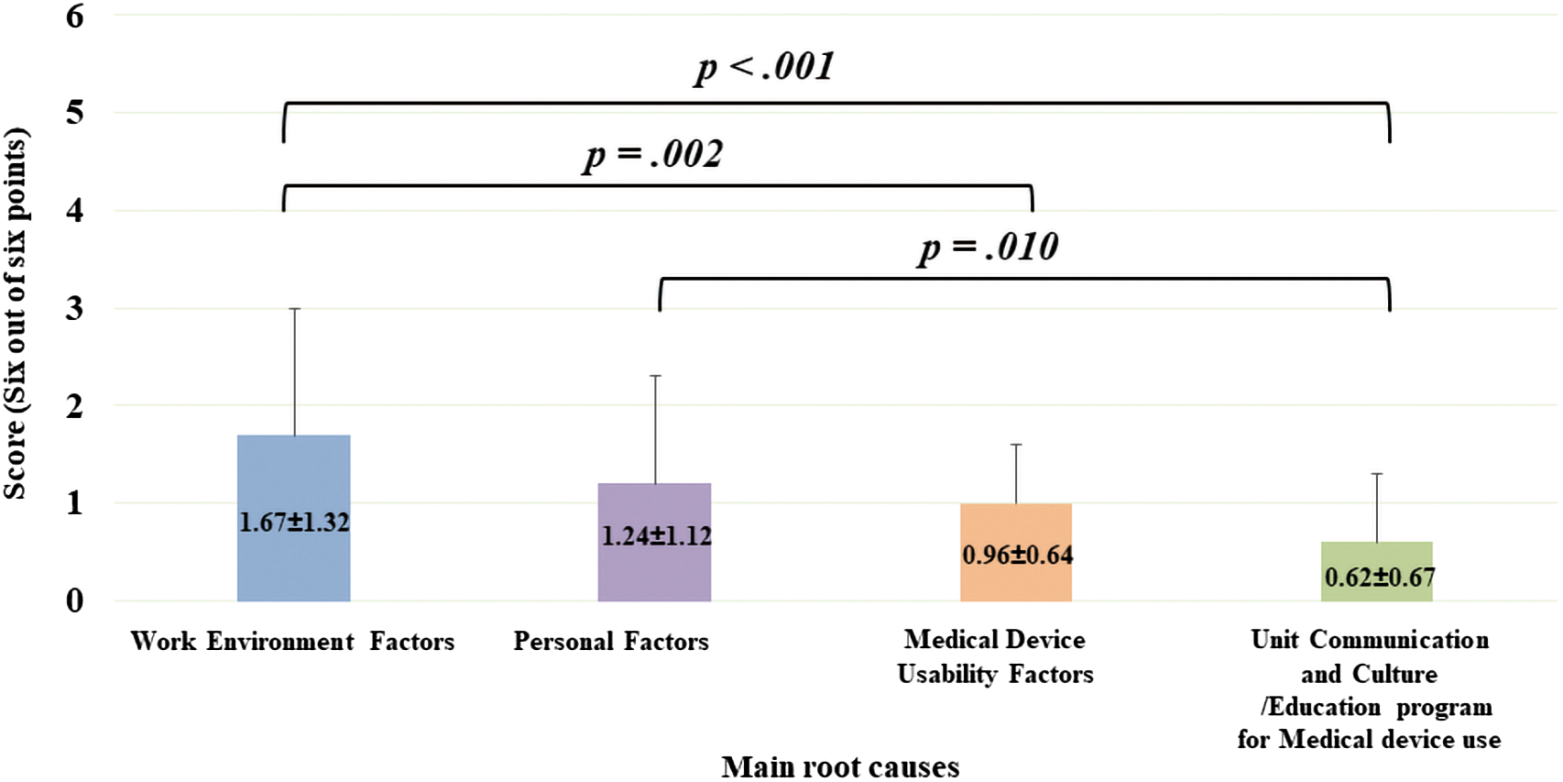
Each root cause score (maximum = 6) is shown as mean ± SD and was compared using one‐way ANOVA.

The internal inconsistency of the questionnaire for RCA was confirmed through Cronbach's α. When Cronbach's α was analyzed by device, the reliability of the Ishikawa diagram was α = .940 in all medical devices (Figure S1). There was a significant difference in scores among the main root causes of NM‐MUEs (*p* < .001). The work environment factors showed the highest score (1.67 ± 1.32), followed by personal factors (1.24 ± 1.12), medical device usability factors (0.96 ± 0.64), and unit communication and cultural/educational programs for medical device use (0.62 ± 0.67, Figure 3).

**FIGURE 4:**
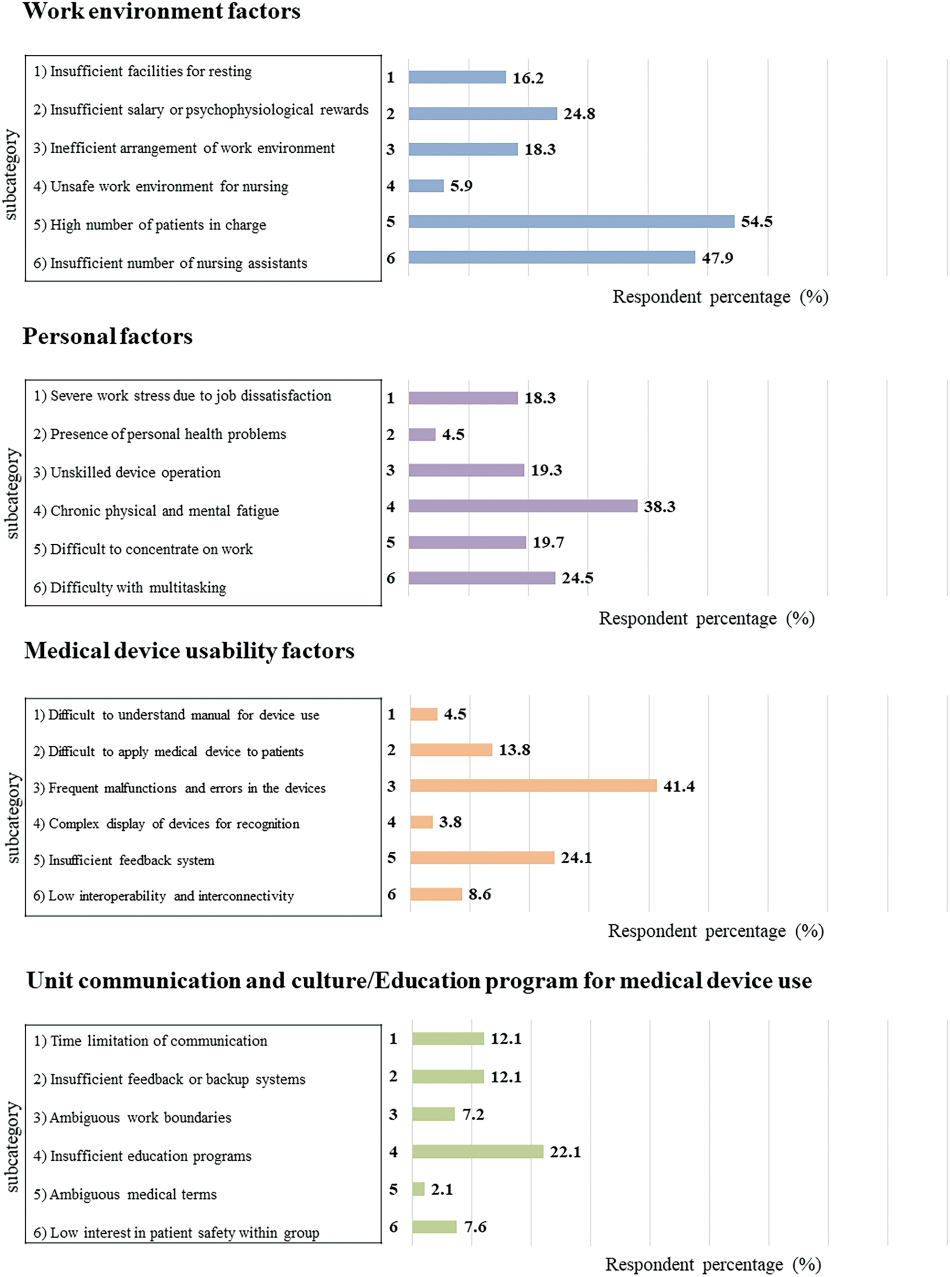
Distribution of root cause subcategories across all medical devices.

Among six subcategories in the work environment factors, the “high number of patients in charge” was indicated as the main cause of NM‐MUEs (54.5%). The “insufficient number of nursing assistants” was also cited as a significant factor (47.9%). Among the subcategories included in the personal factors, “chronic physical and mental fatigue” was the main cause (38.3%). In addition, continuous shift work and changes in daily rhythms due to rotating shifts were cited as causes of fatigue in open questions. The primary cause among medical device usability factors was “frequent malfunctions and errors in the devices” (41.4%). For unit communication and culture/education program factors, the main cause was “insufficient education programs” (22.1%, Figure 4).

### 3.4 Narrative review of the distinct root cause analysis for each medical device

The “frequent malfunctions and errors in the devices,” particularly the frequent detachment of device sensors or leads from patients, was the main cause of NM‐MUEs for SpO_2_ monitors (56.9%) and EKGs (60.3%, Figures S2 and S3). In the case of IV line sets, many subjects indicated that the “Tendency to have difficulty multitasking” among personal factors was also a contributing factor to NM‐MUEs following “chronic physical and mental fatigue” (31.0%, Figure S4). Subjects who selected this option responded that numerous IV lines applied to patients were a primary reason for “difficult to concentrate on work.” In the case of infusion pumps, most subjects reported “frequent malfunctions and errors in the devices” due to old equipment (50.0%, Figure S5) as a primary cause, while others cited “insufficient education programs” lacking proper training and orientation for using the device (39.7%, Figure S5).

## 4 DISCUSSION

According to Heinrich's law, incidents that do not result in actual harm occur much more frequently than incidents that result in harm. As a result, various industries, including the medical field, are making efforts to identify and prevent near‐miss events to avoid actual errors (Joint Commission, 2020).

The FDA reported that from 2010 to 2018, at least 52,196 malfunctions, 9499 injuries, and 269 deaths annually in the United States can be attributed to MUEs (Knisely et al., 2020). Based on this information, it can be concluded that evaluating near‐miss events in MUEs, which occur more frequently than actual events, would be advantageous for identifying factors that could lead to actual MUEs (Knisely et al., 2020; Tanaka et al., 2010).

In this study, we investigated the incidence of NM‐MUEs for five commonly used medical devices in the ICUs. The average annual number of near‐miss events for these five medical devices was 2.11 ± 12.53.

Among them, IV line sets exhibited the highest frequency of near‐miss events. This finding is consistent with the 2023 national data from South Korea, which showed that IV line sets accounted for the largest number of cases reported by regional medical device safety information monitoring centers (National Institute of Medical Device Safety Information, 2023). Additionally, SpO₂ monitors, EKGs, and infusion pumps also exhibited relatively high frequencies compared to previous studies.

Despite the high incidence of medical bed‐associated MUEs in our study, these devices are not included in Korea's medical device safety investigations, suggesting a lack of systematic management and highlighting the need for increased attention. Ideally, interventions should target all devices; however, if this is not feasible, prioritizing IV line sets is reasonable given their high incidence and the specific operational characteristics of each hospital.

Interestingly, we observed a high degree of variability in the frequency of NM‐MUEs. Further analysis revealed a positive correlation between nursing experience and the frequency of near‐miss event reports. This trend is consistent with previous studies, suggesting that the lower reporting rates by nurses with shorter career experience may be due to their limited exposure to the term “near‐miss error” until they began working in the hospital (Lee, 2021).

The lack of adequate care time for patients is a well‐recognized factor in MUE occurrences (Mattox, 2012; Seki & Yamazaki, 2006). Consistent with previous research, our study also identifies a high patient load combined with insufficient staff support as a primary factor leading to inadequate patient care time. While a nurse‐to‐patient ratio of less than 2:1 is generally recommended, our study shows that each ICU nurse in our hospital is responsible for an average of 3.1 patients. This result aligns with a Korean study that reported an average nurse‐to‐patient ratio of approximately 1:2.7 across South Korea (Kwak et al., 2014). Both domestic and international literature recommend maintaining a nurse‐to‐patient ratio of 1:1 or 1:2 for ICUs (Marshall et al., 2017; Neuraz et al., 2015). However, South Korea's medical insurance system presents significant challenges in achieving this goal. Addressing this issue requires national attention and support (Kim et al., 2017; Stimpfel et al., 2022).

Among personal factors, physical and mental fatigue caused by continuous shift work and high labor intensity was found to significantly contribute to MUEs. These findings are consistent with previous studies reporting a higher frequency of NM‐MUEs in individuals experiencing severe fatigue than in those who are not (Caruso, 2014; Stimpfel et al., 2022). To achieve this, individuals must ensure adequate rest, including sufficient sleep, before each shift. Adjusting work schedules to allow appropriate intervals between shifts can effectively reduce stress and promote mental recovery (Caruso, 2014). Institutional support, including adequate staffing, is essential for achieving these goals (Stimpfel et al., 2022).

When reviewing device‐related causes in detail, unexpected electrode detachment in SpO_2_ monitors and EKGs was identified as a major cause of MUEs. Therefore, improving the adhesive strength of components in patient vital signs monitoring devices or exploring alternative monitoring methods is necessary. Infusion pumps are known to have a high incidence of often fatal MUEs. The FDA has identified various contributing factors (Food and Drug Administration, 2017). In our study, alarm‐related issues were the primary cause, including failures to activate, persistence despite reset attempts, and malfunctions due to confusing warning messages on outdated equipment. Given their association with aging equipment, immediate replacement of outdated infusion pumps is recommended. Based on the results of our survey, it is advisable to implement educational programs for novice nurses covering how to handle device operation, alarm messages, and alarming situations. Moreover, simulations using mannequins or dummies are essential (Eisold et al., 2015). These simulations should be conducted in settings resembling actual clinical environments or familiar surroundings, enabling direct practice of medication administration (Eisold et al., 2015).

There are several considerations when interpreting the results of this study. First, as this study was conducted at a single center, it is difficult to generalize the findings to other hospitals or healthcare settings. The specific environment and procedures of one institution could influence the results, potentially limiting the applicability of the findings to other settings. Second, although the survey was prospective, the number of events was assessed based on recall memory, resulting in discrepancies between reported and actual frequencies. Third, as the study focused on exploring causes through RCA, it does not offer insights into the results of actual interventions and improvements. Also, Ishikawa diagrams are suitable for addressing healthcare‐related issues owing to their easy design and flexibility. When a diagram oversimplifies a problem, it becomes difficult to prioritize the most important problem. In addition, the problems and causes identified in this paper can be subjectively evaluated based on the characteristics of the institution. To overcome the mentioned challenges, we endeavored to utilize existing data from our hospital to select medical devices. We focused on minimizing these issues through interviewing healthcare practitioners and professionals and expert conferences.

## 5 CONCLUSION

This study provides valuable insights into the root causes of NM‐MUEs in ICUs. The findings demonstrate that NM‐MUEs are influenced by both systemic and individual factors, with high patient load, insufficient nursing support, and chronic fatigue being the most significant contributors. Frequent malfunctions in medical devices, particularly IV line sets, SpO_2_ monitors, and EKGs, also played a critical role. The study underscores the need for targeted interventions, including updating equipment, improving nurse‐to‐patient ratios, and enhancing educational programs. These measures are essential to mitigate the risks of MUEs and improve patient safety. Future studies should focus on broader hospital settings to further explore the prevalence and prevention of MUEs and implement interventions based on RCA findings. Additionally, addressing work fatigue through better staffing and rest facilities will be crucial in reducing NM‐MUEs.

## AUTHOR CONTRIBUTIONS

Conceptualization: Bo Hae Kim. Methodology: Su Mi Seong, Bo Hae Kim. Data curation: Hyeop Oh. Analysis: Su Mi Seong. Funding acquisition: Bo Hae Kim. Investigation: Su Mi Seong. Project administration and Supervision: Bo Hae Kim, Bum Sun Kwon. Resources and Software: Su Mi Seong. Validation: Jae Suk Park, Su Hyun Bae, Ki Chang Nam, Sung Yun Park, Bum Sun Kwon, Ki Chang Nam, Sung Yun Park, and Bo Hae Kim. Visualization: Su Mi Seong, Hyeop Oh. Writing original draft: Su Mi Seong. Review and editing: Bo Hae Kim.

## CONFLICT OF INTEREST STATEMENT

The authors declare that there is no conflict of interest.

## Supporting information


**Figure S1.** Cronbach's alpha of each medical devices.
**Figure S2.** The root causes in the subcategories of SpO_2_ monitors.
**Figure S3.** The root causes in the subcategories of the IV line sets.
**Figure S4.** The root causes in the subcategories of the infusion pump.
**Figure S5.** The root causes in the subcategories of the EKG.
**Figure S6.** The root causes in the subcategories of the medical beds.
