# Supplementary material for: Incidence and root cause analysis of near‐miss events in medical device use errors in intensive care units using Ishikawa diagram
Source: Jpn J Nurs Sci. 2025 Sep 15;22(4):e70024. doi: 10.1111/jjns.70024 (PMC12434650; doi:10.1111/jjns.70024)
Supplement: Supplementary file 1 — Figure S1. Cronbach's alpha of each medical devices. Figure S2. The root causes in the subcategories of SpO2 monitors. Figure S3. The root causes in the subcategories of the IV line sets. Figure S4. The root causes in the subcategories of the infusion pump. Figure S5. The root causes in the subcategories of the EKG. Figure S6. The root causes in the subcategories of the medical beds. [file JJNS-22-e70024-s001.docx]

**Supplementary material**

Supplementary figure 1. Cronbach's alpha of each medical devices.


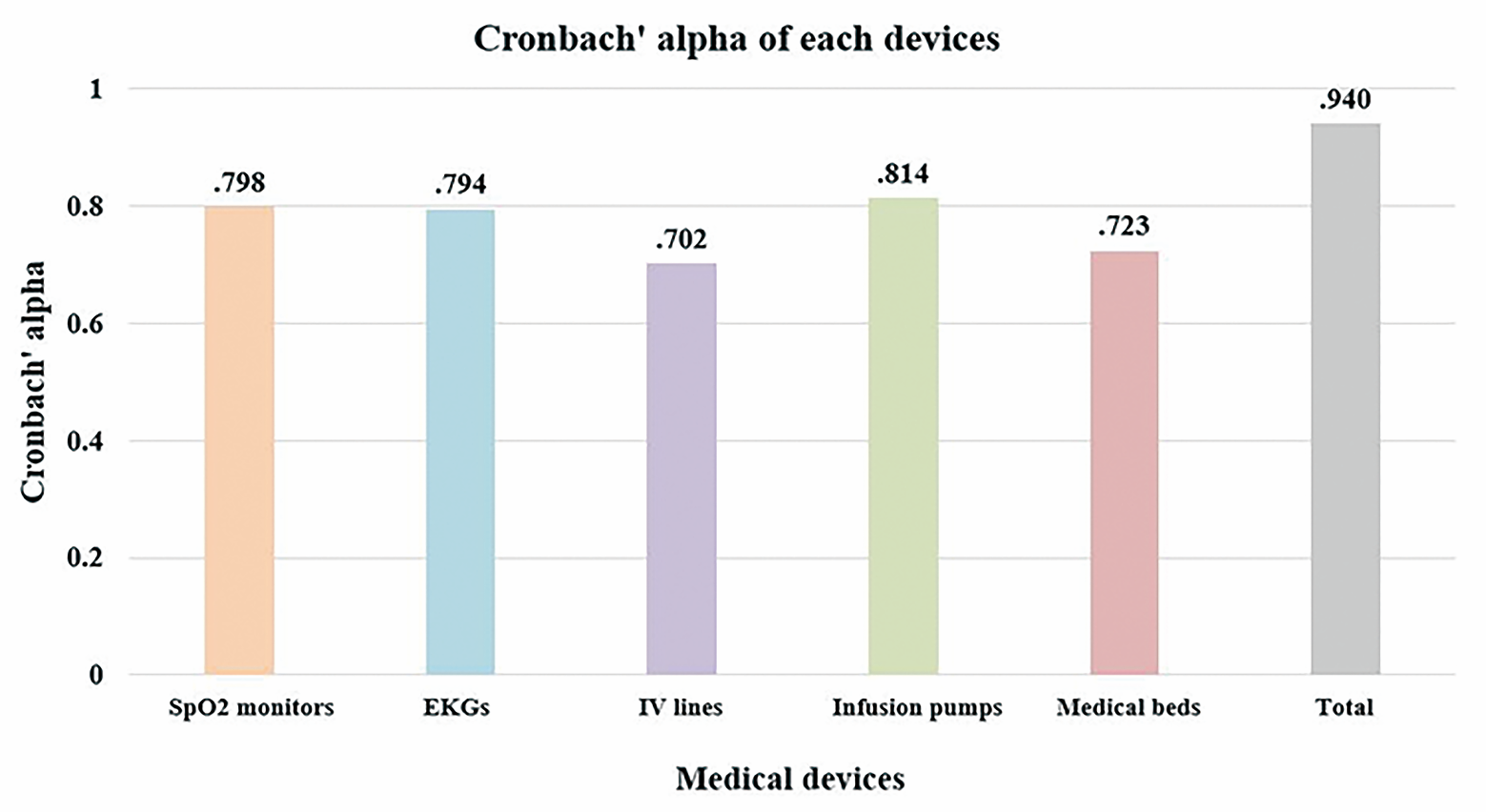


The Cronbach’s alpha values were α = .940 for all five medical devices, followed by α = .814 for infusion pumps, α = .798 for SpO2 monitors, α = .794 for EKGs, α = .723 for medical beds, and α = .702 for IV lines.

Supplementary figure 2. The root causes in the subcategories of SpO2 monitoring.

**
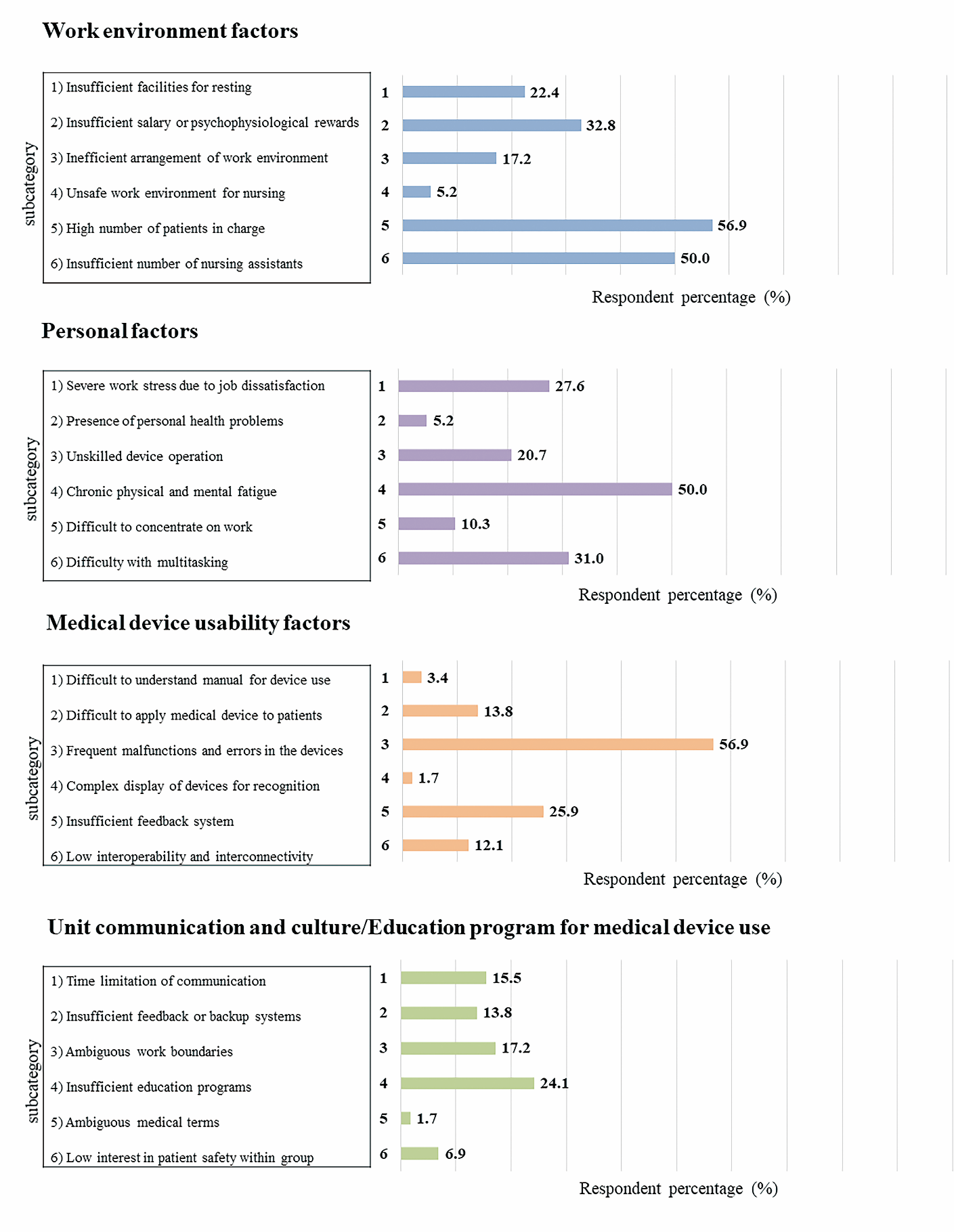
**

Supplementary figure 3. The root causes in the subcategories of the IV line sets.

**
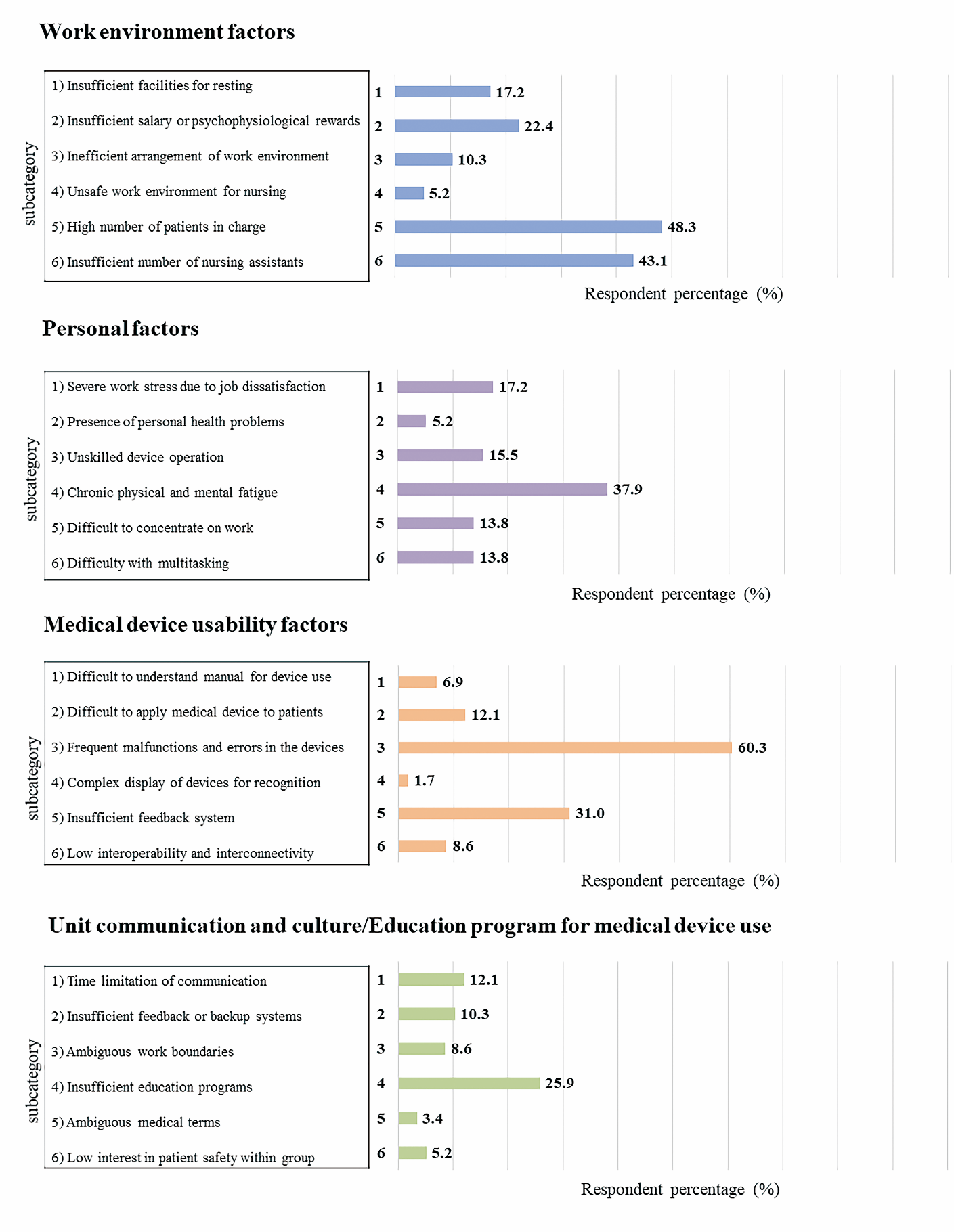
**

Supplementary figure 4. The root causes in the subcategories of the infusion pump.

**
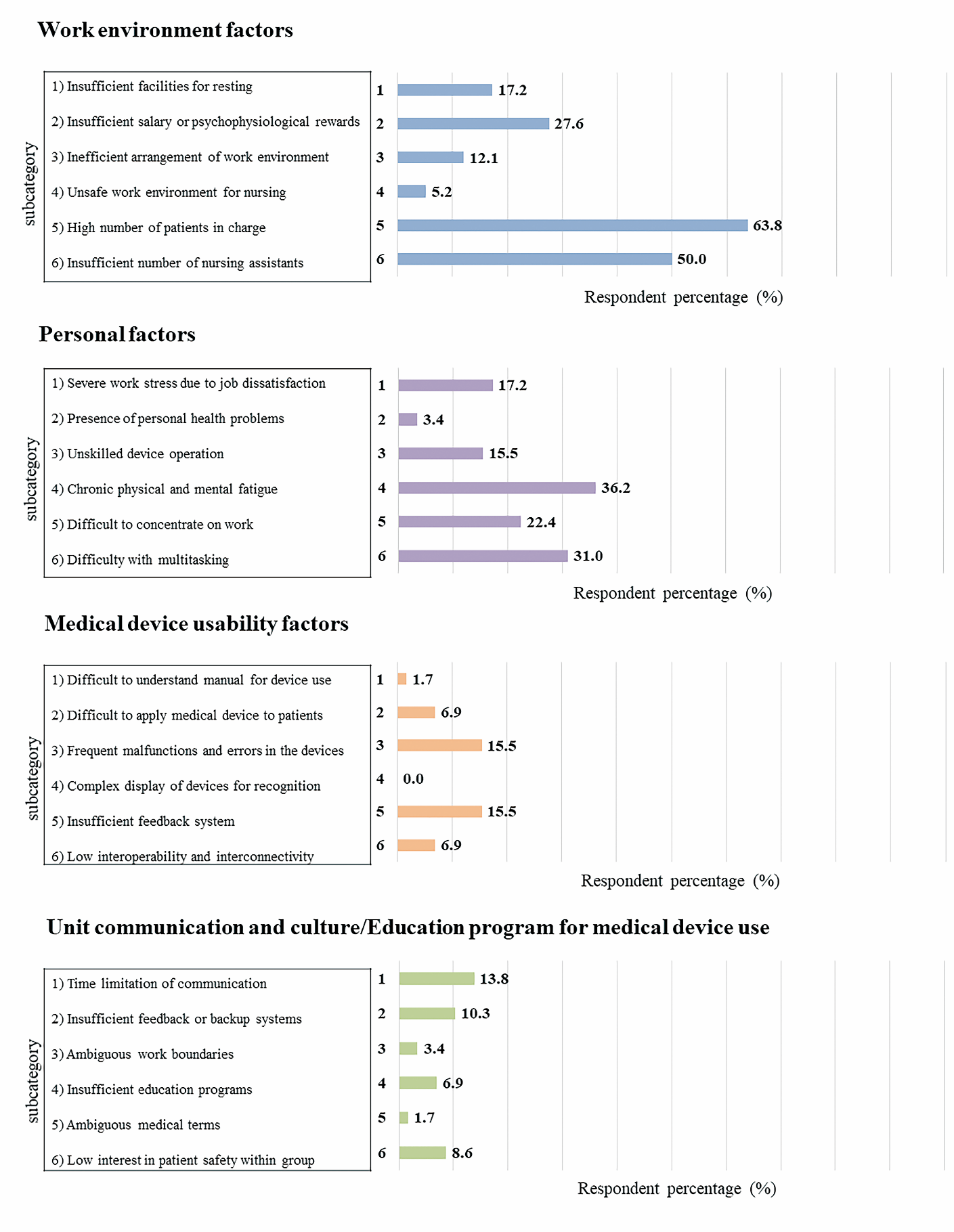
**

**Supplementary figure 5. The root causes in the subcategories of the EKG.**

**
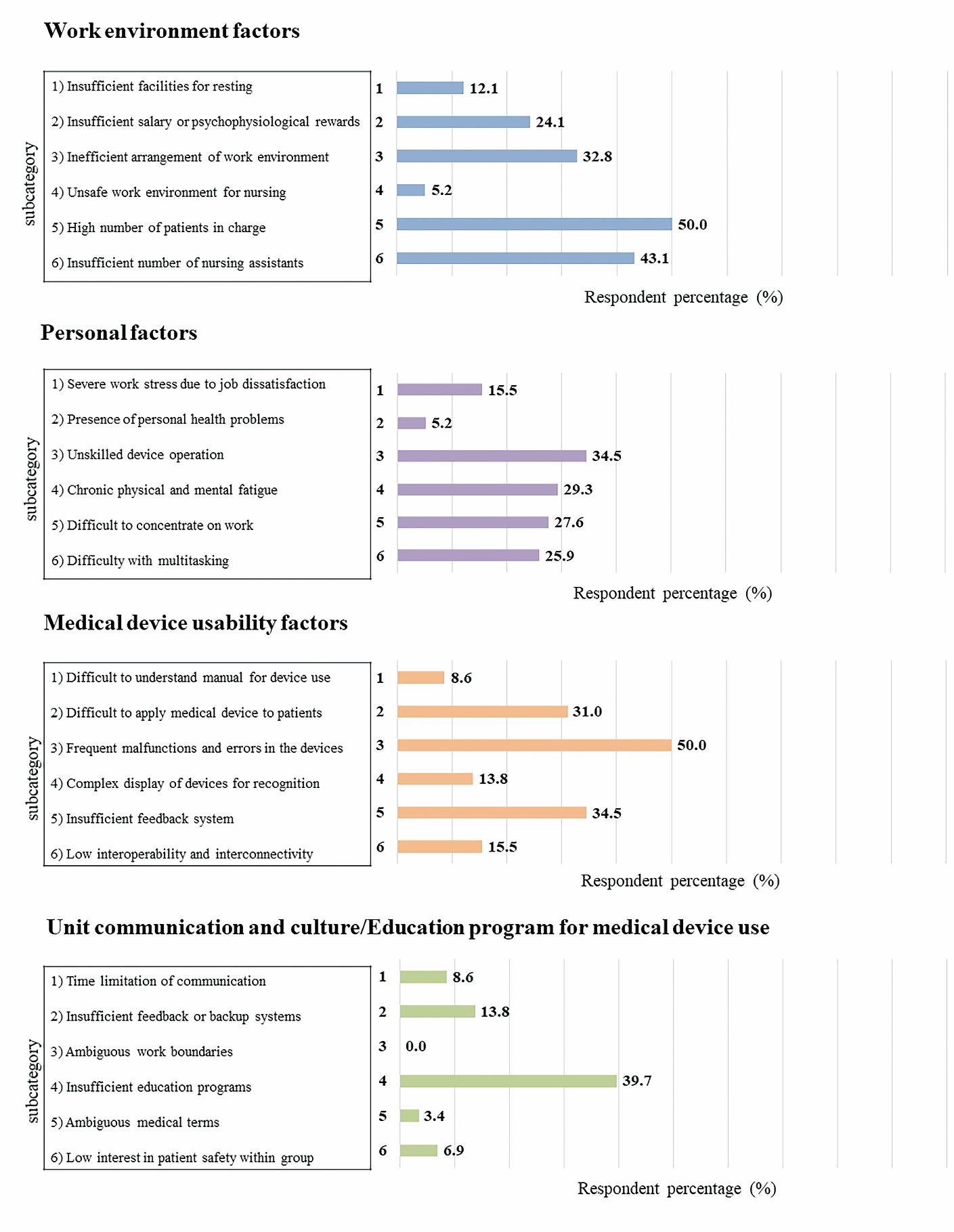
**

**Supplementary figure 6. The root causes in the subcategories of the medical beds.
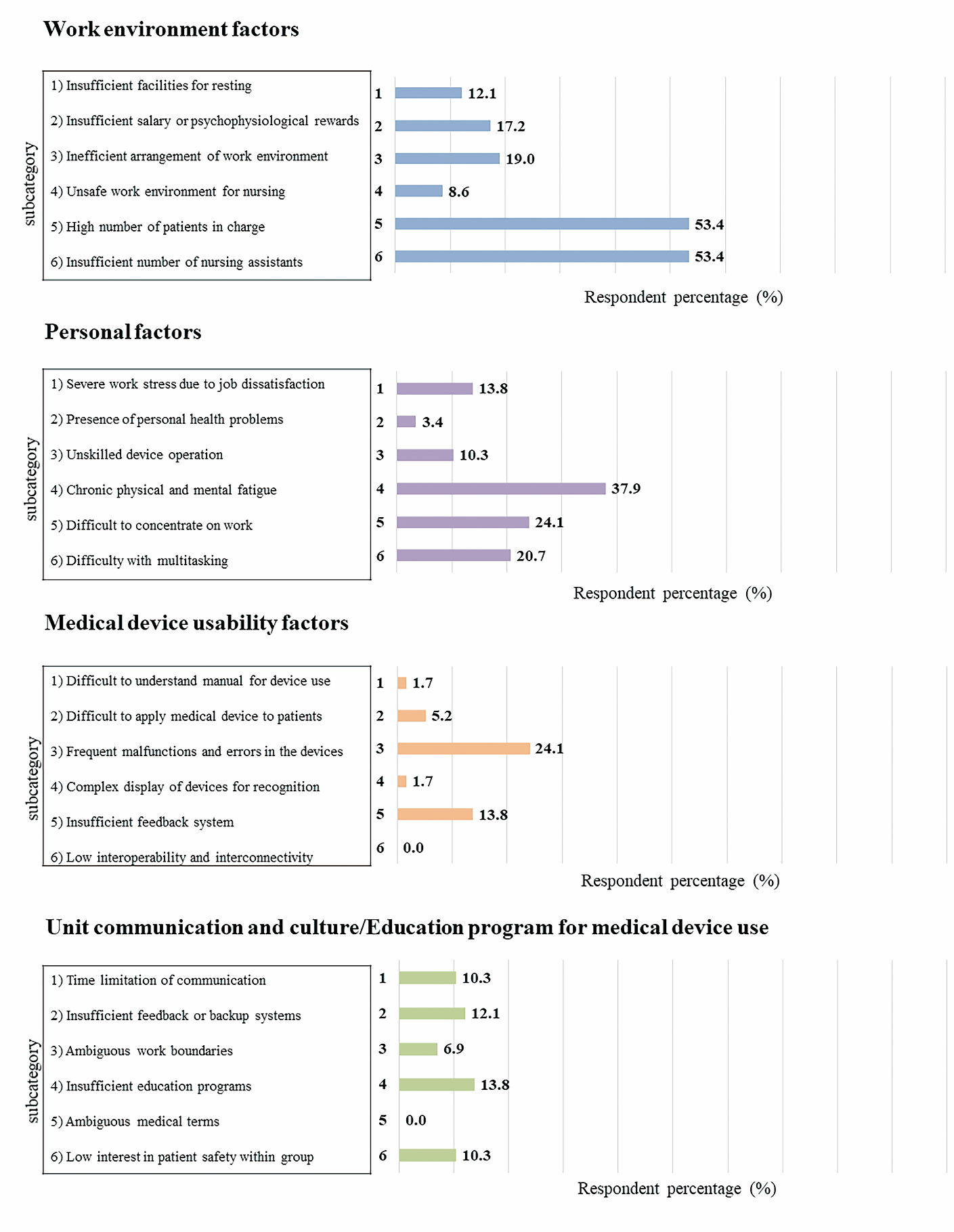
**
